# Chimeric Antigen Receptor T-Cell Therapy in Lung Cancer: Potential and Challenges

**DOI:** 10.3389/fimmu.2021.782775

**Published:** 2021-11-01

**Authors:** Bu-Fan Xiao, Jing-Tao Zhang, Yu-Ge Zhu, Xin-Run Cui, Zhe-Ming Lu, Ben-Tong Yu, Nan Wu

**Affiliations:** ^1^ Key Laboratory of Carcinogenesis and Translational Research (Ministry of Education), Department of Thoracic Surgery II, Peking University Cancer Hospital & Institute, Beijing, China; ^2^ Department of Thoracic Surgery, The First Affiliated Hospital of Nanchang University, Nanchang, China; ^3^ Key Laboratory of Carcinogenesis and Translational Research (Ministry of Education/Beijing), Laboratory of Biochemistry and Molecular Biology, Peking University Cancer Hospital & Institute, Beijing, China

**Keywords:** chimeric antigen receptor, T cell, immunotherapy, lung cancer, engineering strategy

## Abstract

Chimeric antigen receptor T (CAR-T) cell therapy has exhibited a substantial clinical response in hematological malignancies, including B-cell leukemia, lymphoma, and multiple myeloma. Therefore, the feasibility of using CAR-T cells to treat solid tumors is actively evaluated. Currently, multiple basic research projects and clinical trials are being conducted to treat lung cancer with CAR-T cell therapy. Although numerous advances in CAR-T cell therapy have been made in hematological tumors, the technology still entails considerable challenges in treating lung cancer, such as on−target, of−tumor toxicity, paucity of tumor-specific antigen targets, T cell exhaustion in the tumor microenvironment, and low infiltration level of immune cells into solid tumor niches, which are even more complicated than their application in hematological tumors. Thus, progress in the scientific understanding of tumor immunology and improvements in the manufacture of cell products are advancing the clinical translation of these important cellular immunotherapies. This review focused on the latest research progress of CAR-T cell therapy in lung cancer treatment and for the first time, demonstrated the underlying challenges and future engineering strategies for the clinical application of CAR-T cell therapy against lung cancer.

## 1 Introduction

Lung cancer is one of the most frequently occurring malignant tumors worldwide and is characterized by a substantially high malignancy and poor prognosis (1). According to the latest global cancer statistics, lung cancer remains the leading cause of cancer-related deaths worldwide (2). Lung cancer can be histologically classified into two main subtypes: small-cell lung carcinoma (SCLC) and non-small-cell lung carcinoma (NSCLC) (3). NSCLC accounts for approximately 85% of diagnosed lung cancer cases and can be further divided into adenocarcinoma, squamous cell carcinoma, and large cell carcinoma (4, 5).

The present therapeutic measures for NSCLC primarily include surgical resection, chemoradiation, molecular-targeted therapy, and immunotherapy (6). The surgical resection procedure was based on the TNM stage of NSCLC patients. Conventional or stereotactic radiotherapy is applicable to patients with surgically unresectable NSCLC (7). Platinum-based double-agent combination chemotherapy is generally accepted as the standard chemotherapy regimen for NSCLC (8). Neoadjuvant chemotherapy is applied preoperatively to downgrade the cancer stage, whereas adjuvant chemotherapy is administered postoperatively, primarily involving cisplatin-based combination regimens (7). The primary molecular-targeted therapies include epidermal growth factor receptor tyrosine kinase inhibitors (EGFR-TKIs), anti-EGFR monoclonal antibodies, fusion gene ALK and ROS1 inhibitors, and anti-vascular endothelial growth factor receptor monoclonal antibodies (9–12). Combined therapy with multiple immune checkpoint inhibitors, such as a combination of nivolumab and ipilimumab, has been shown to achieve better response rates than monotherapy (13, 14).

Non-surgical treatment involving systemic chemotherapy plus radiotherapy is the mainstream procedure for SCLC patients because metastases occur when SCLC is newly diagnosed. Etoposide-platinum and topotecan are the standard first-line and second-line regimens for SCLC patients, respectively (15, 16). Although SCLC is very sensitive to chemotherapy, many SCLC patients relapse due to the clinical development of chemoresistance. Moreover, nivolumab was the first FDA-approved immunotherapy agent for SCLC treatment (17). Several small molecular inhibitors, including PARP inhibitors, have also been demonstrated to exert anti-tumor activity in SCLC in clinical trials (18, 19). However, due to the heterogeneity of tumors, it is imperative to explore effective novel therapies.

**Figure 1:**
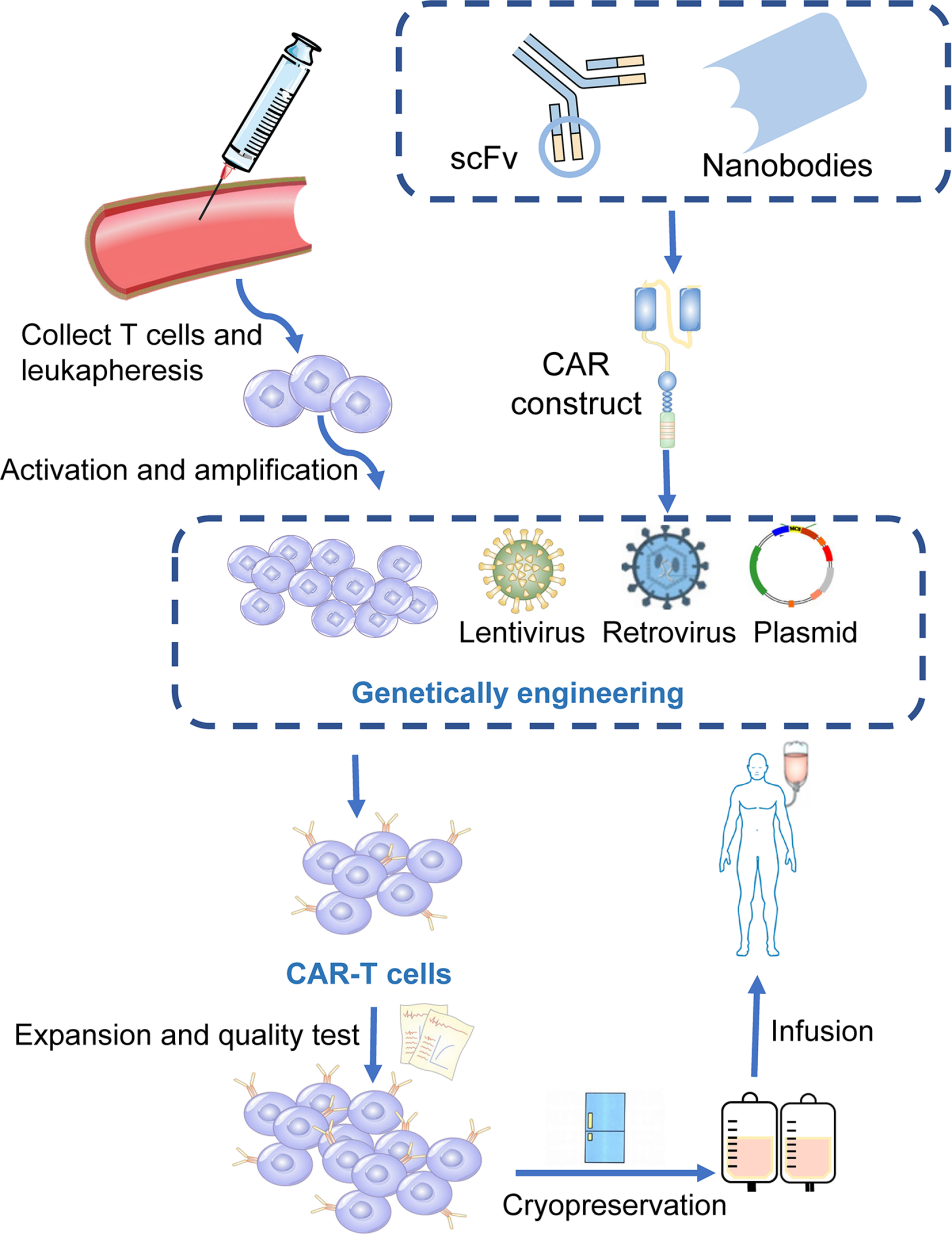
Manufacturing procedures of CAR-T cells. T cells are firstly collected from the peripheral blood of the patients. The activated and amplified T cells are genetically engineered with CAR structure *via* retroviral, lentivirus or other vectors. CAR-T cells are then expanded *ex vivo* and a quality control procedure is applied. Finally, those modified T cells were infused back into the patients.

Chimeric antigen receptors (CARs) are engineered receptors that can enable modified T cells to recognize and kill tumor cells expressing a tumor-specific antigen (20). CAR-T cells contain two sections: autologous T cells separated from the peripheral blood of patients and integration of CARs into T cells through genetic engineering in the laboratory. Patient’s T cells are extracted, isolated, and genetically engineered to express a CAR on their surface, targeting tumor-specific antigens of cancer cells. The modified CAR-T cells are amplified *in vitro* and then infused back into the patients **(** **Figure 1** **)** (21). Subsequently, CARs can identify and bind to specific antigens expressed on cancer cells and consequently eliminate and kill cancer cells (22, 23).

CAR-T cell therapy is an emerging method against hematological malignancies and has demonstrated satisfactory curative effects, which is a substantial breakthrough in adoptive cell therapy (24, 25). CAR-T cells targeting CD19 have become a leading engineered T-cell therapy strategy against relapsed or refractory acute lymphocytic leukemia and B-cell non-Hodgkin lymphoma (26, 27). Yescarta (axicabtagene ciloleucel) and Kymriah (tisagenlecleucel) are currently approved to treat B-cell-derived malignancies, with response rates greater than 80% (28, 29). Recently, Tecartus (brexucabtagene autoleucel) has also been approved for the treatment of adult mantle cell lymphoma (30, 31). However, only targeting CD19 did not show considerable efficacy in most refractory multiple myeloma (MM) patients, partly due to the lower expression of CD19 on the cell surface of myeloma, and there is no FDA-approved CAR-T cell therapy against it (22, 32, 33). Clinical trials have indicated that CD269 (B cell maturation antigen, BCMA) and CD138 (also known as syndecan 1) molecules, which are mostly expressed in mature B cells or plasma cell surfaces, could exert substantial anti-MM activity (34–36). The unprecedented achievements of CAR-T cell therapy in hematological malignancies have also improved the use of CAR-T cells in various solid tumors.

### 1.1 The Design and Development of CAR Structure

**Figure 2:**
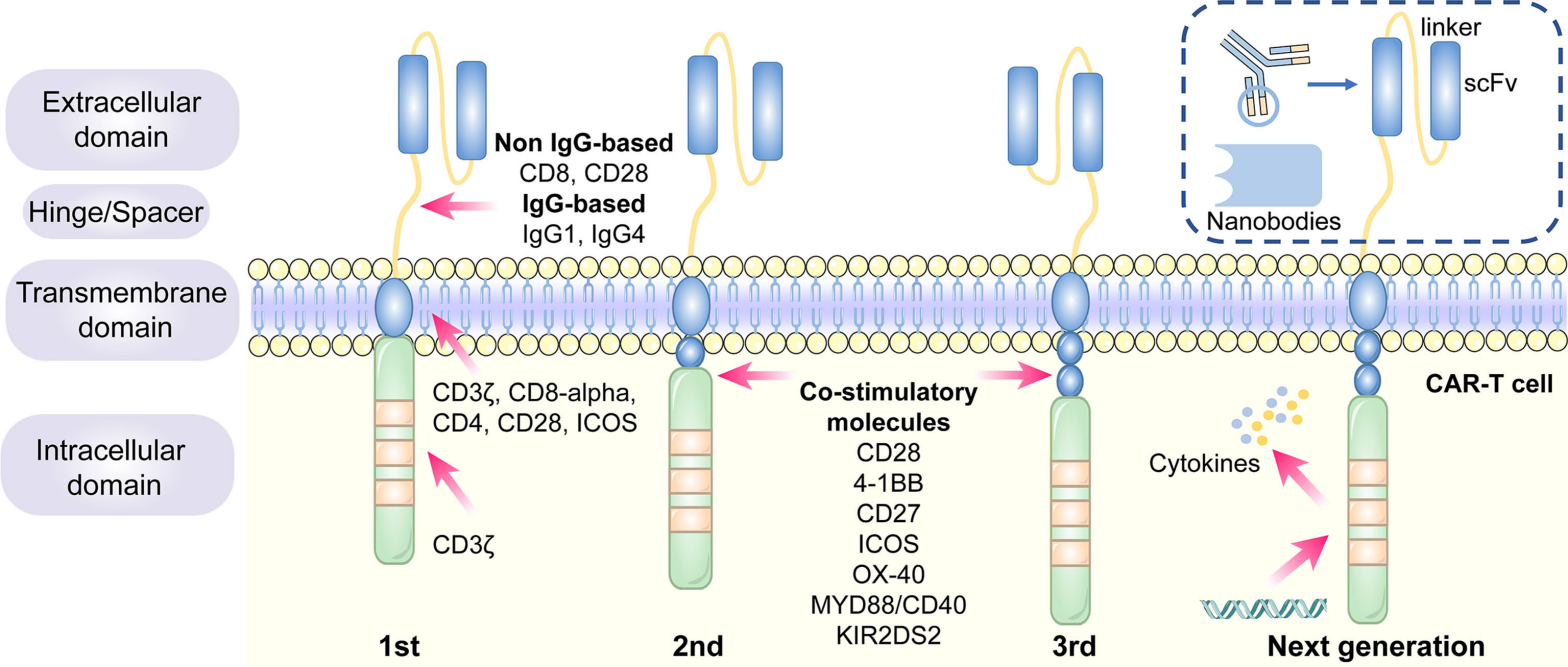
The structure and evolution of CAR-T cells from the first generation to the fourth generation. The CAR-T cells are consisted of extracellular tumor antigen binding domains (scFv, nanobodies), hinge regions, transmembrane regions and intracellular signaling domains. Different generations of CAR structures are primarily characterized by distinct intracellular signaling domains. The first generation of CAR-T cells only contain a CD3ζ intracellular signaling domain, with less persistence and efficacy in clinical practice. The second or third generation of CAR-T cells include one or more costimulatory molecules, and the next generation of CAR-T cells are engineered to express cytokines, which greatly improve their competence to eliminate the tumor cells.

CARs are artificial fusion proteins that comprise four major parts: extracellular antigen recognition and binding domains, spacer/hinge domains, transmembrane domains, and intracellular signaling domains (37, 38). Every component of the CAR structure has unique properties and has evolved to optimize the CAR function (39). The extracellular domains are responsible for recognizing and binding the targeted tumor-specific antigens, whereas intracellular signal domains primarily induce T-cell proliferation and corresponding signal transduction **(** **Figure 2** **)** (40). Recently, armored CAR-T cells have been engineered to overcome immunosuppressive tumor microenvironment (TME) (41). Engineered CAR-T cells can secrete various cytokines such as IL-12, chemokines, or co-expressing immunomodulatory ligands to alter the inhibitory microenvironment in the TME and support CAR-T cell function (20).

#### 1.1.1 Antigen Recognition and Binding Domains

The single-chain variable fragment (scFv) is derived from the variable heavy and variable light chains of a monoclonal antibody connected by a flexible linker (42). It is the major component of the extracellular antigen recognition and binding moieties, which can effectively recognize tumor antigen targets in a major histocompatibility complex (MHC)-independent manner and trigger CAR downstream signaling and CAR-T cells (43). The scFv sequences determine the specificity and binding affinity of the targeted antigens of the CAR (44). The high affinity of scFv has been reported to result in on-target, off-tumor toxicity, and severe cytokine release syndrome (45). Moreover, scFv can be designed to bind to soluble ligands, such as transforming growth factor-beta (TGF-β), contributing to the conversion of the immunosuppressive role of TGF-β (46). Single-domain antibodies (known as nanobodies or VHHs), whose variable regions only contain heavy chains instead of light chains, are stable camelid-derived single-domain antibodies (47). They are smaller in size and have a similar affinity to traditional scFv; however, they avoid the shortcomings of traditional scFv, such as low folding efficiency and tendency to aggregate (48, 49). In addition, cytokines (50), ligands (51–54) and antigen recognition peptides (adnectins and designed ankyrin repeat proteins) could be applied as an option for antigen recognition and binding regions of CARs (55, 56).

#### 1.1.2 Hinge Domains

The length of the hinge regions can be adjusted to optimize the distance between CAR-T cells and targeted tumor cells, ensuring the folding efficiency of CAR scFv and providing a flexible and persistent connection for CAR signal transduction (57). In addition, the domains also augment the binding affinity of CAR-T cells and targeted cells (38). Hinge domains play a crucial role in regulating the expression and transport efficiency of CAR and the definition of the CAR signaling threshold (57). The spacer domains enable the CAR to access target epitopes that are otherwise sterically inaccessible (58). They can also be used to modulate synaptic cleft distances, as distal membrane antigen epitopes commonly require shorter spacers, whereas proximal membrane antigen epitopes require longer spacers (58, 59). Non-IgG-based spacers, including CD8 and CD28, and IgG-based spacers, such as IgG1 or IgG4, have been proven to be equally effective and are utilized in the construction of CAR hinge domains (58, 60). The spacers containing Fc domains must be changed after recognizing the targeted antigens, in case of *in vivo* interactions with cells expressing Fc gamma receptors that result in off-target activation of CAR-modified T cells or impaired antitumor efficacy (61).

#### 1.1.3 Transmembrane Domains

The transmembrane domains serve as anchors to connect the extracellular antigen-binding domain to the cell membrane and transduce extracellular antigen-recognition signals to the intracellular domains (38, 58). They primarily originate from type I transmembrane proteins, including CD3ζ, CD8-alpha, CD4, or CD28 (20, 62). The stability and function of CARs are associated with transmembrane domains (38). Bridgeman et al. reported that CARs containing the CD3ζ transmembrane domain can form a complex with endogenous T cell receptor (TCR), and subsequently, may induce T cell activation (63). *In vivo* studies indicated that CD8-alpha resulted in lower levels of inflammatory cytokines and T-cell activation-induced death than CD28 (64). CD28 is currently the most stable transmembrane domain (39). Third-generation CAR T cells carry a B7-family inducible costimulator (ICOS) transmembrane domain (65). The persistence and anti-tumor activity of CAR-T cells is substantially promoted when the ICOS transmembrane domain is connected to an ICOS intracellular domain (62).

#### 1.1.4 Intracellular Signaling Domains

The endodomains normally comprise a CD3ζ transducer, and one or more co-stimulatory signaling molecules such as CD28, 4-1BB (CD137), CD27, ICOS, OX-40, MYD88/CD40, and KIR2DS2 (66). This design pattern further prolongs the survival time and promotes the proliferation and antitumor activities of CAR-T cells (38, 67, 68). CD28 and 4-1BB, fused to the intracellular CD3ζ domain, are the most extensively studied and intensively applied co-stimulatory molecules (69). However, their clinical efficacy is far from each other. CAR-T cell therapy based on 4-1BB costimulatory domain is generally admitted to have more superior clinical efficacy, because 4-1BB costimulatory domain could ameliorate the exhaustion mediated by CAR signaling (70, 71). CAR-T cell product based on CD28 costimulatory domain initiates faster antitumor property, while compared with 4–1BB costimulatory domain, it is less persistent since fewer central memory T cells are formed (72) (**Table 1**). Additionally, CAR-T cells, incorporated two costimulatory molecules, such as ICOS and 4-1BB, have showed tremendous efficacy in preclinical mouse models (62, 73).The other co-stimulatory signaling molecules, including CD27 (74, 75), OX-40 (76, 77), MYD88/CD40 (78) and KIR2DS2 (79) have demonstrated promising efficacy in preclinical models but have not been tested in clinical trials.

**Table 1:** Comparison of properties of different costimulation 4-1BB versus CD28 in CAR-T cell.

Property	4-1BB	CD28
Expansion ability	Low	High
Anti-tumor response	Persistent	Rapid
Susceptibility to exhaustion	Low	High
Phenotype formation	Memory phenotype	Effector phenotype
Metabolic type	Fatty acid oxidative metabolism	Glycolytic metabolism
Overall efficacy	Superior	Inferior

### 1.2 The Generation of CAR−T Cells

Different generations of CAR structures, characterized by distinct intracellular signaling domains, have been designed to improve the safety and efficacy of CAR-T cell therapy against various cancers (80). First-generation CAR-T cells only contain one intracellular signaling domain, CD3ζ, with less impressive clinical efficacy for the lack of persistence and proliferative activity (38). Inclusion of the costimulatory molecules equipped with second-generation CAR-T cells with the necessary signals for activation considerably prolonged the survival time of CAR-T cells and improved clinical outcomes in cancer patients (81). Third-generation CAR-T cells aggrandize a costimulatory molecule compared with second-generation CAR-T cells, consisting of CD3ζ and two costimulatory molecules (CD27, CD28, 41BB, ICOS, OX-40, etc.), further augmenting and enhancing their competence to clear tumor cells (82, 83). In particular, the fourth generation of CAR-T cells known as T cells redirected for universal cytokine-mediated killing (TRUCK), which can recruit nuclear factor of activated T cells (NFAT) to induce the release of cytokines IL-12 IL-15 and granulocyte–macrophage colony-stimulating factor (84). The anti-tumor activity of the fourth generation of CAR-T cells is enhanced by overcoming the immunosuppressive effect of the TME **(** **Figure 2** **)**. The fifth-generation CAR-T cells, which is proposed to remove the TCR alpha and beta chains through gene editing technology, avert the risk of graft-vs.-host disease, and manufacture “off the shelf” products, are still under investigation (85).

Although the structure of CARs is constantly evolving to promote efficacy and diminish the cytotoxic effects of CAR-T cell therapy, second-generation CAR-T cells still remain the mainstay of clinical application (86).

### 1.3 NSCLC and SCLC−Associated Antigens for CAR−T Cell Therapy in Preclinical Studies

CAR-T cell therapy has emerged as a novel approach to adoptive cell immunotherapy in recent decades. In solid cancers, it is more complex to construct CAR-T cells because it is difficult to identify tumor-specific antigens to be targeted. Several surface antigens have already been evaluated in preclinical studies as potential CAR-T cell therapy targets. Thereafter, we provide detailed descriptions of several novel targets.

#### 1.3.1 Mesothelin (MSLN)

MSLN, a tumor differentiation antigen with the low expression on normal mesothelial cells, is overexpressed in a wide range of solid cancers, including lung cancer, mesothelioma, and pancreatic carcinoma; therefore, it could be used as a potential target (87, 88). High expression of MSLN is commonly correlated with negative clinical outcomes in NSCLC (67). In *ex vivo* experiments, MSLN-targeted CAR-T cells exerted substantial inhibitory effects on cancer cell proliferation and invasion (89). The efficiency of MSLN-targeted CAR-T cell therapy has been assessed in subcutaneous mouse lung cancer models (90). A slower growth rate of tumor size was observed in the tail vein injection of MSLN-targeted CAR-T cells (89). In summary, MSLN-targeted CAR-T cells could be feasible for MSLN-positive cancers, such as NSCLC.

#### 1.3.2 EGFR

EGFR belongs to the HER/ErbB family of receptor tyrosine kinases that transduces extracellular growth signaling into the cells (91). More than 60% of NSCLC patients harbor activating EGFR mutations, contributing to the overexpression of EGFR, making it possible to target EGFR as a treatment for CAR-T cell therapy against NSCLC (91). EGFR-CAR T cells were found to exhibit greater cytotoxic activity *in vitro* (92). In nude mouse subcutaneous xenografts, EGFR-CAR T cells dramatically decreased tumor size and volume (93). The above results indicate that EGFR-targeted CAR-T cell therapy could be applied to NSCLC patients in the future (94).

#### 1.3.3 Receptor Tyrosine Kinase-Like Orphan Receptor 1 (ROR1)

ROR1 is a crucial oncofetal glycoprotein that can sustain pro-survival and pro-apoptotic signaling in lung adenocarcinomas (95, 96). It has been proposed as a targeted antigen in CAR-T cell therapy as the overexpression of ROR1 protein has been observed in various malignancies, including lung cancer (97, 98). ROR1-CAR T cells maintained their anti-tumor activity, cytokine secretion, and proliferation in NSCLC models *in vitro and in vivo* (97, 99). Carolina et al. demonstrated the safety and function of second-generation ROR1 CAR-T cells in macaques (100).

#### 1.3.4 Mucin-1 (MUC1) and Prostate Stem Cell Antigen (PSCA)

Aberrant high expression of MUC1 regulates the expression of programmed death-ligand 1 (PD-L1) in cancer cells, which could prevent cancer cells from being cleared by the immune system (101, 102). PSCA, a glycosylphosphatidylinositol (GPI)-anchored cell surface protein, belongs to the Thy-1/Ly-6 family (103). MUC-CAR T cells and PSCA-CAR T cells identify and eliminate PSCA+ or MUC1+ NSCLC cells, respectively, *in vitro* (104). PDX mouse subcutaneous models generated from NSCLC patients whose tumors only express PSCA or both PSCA and MUC1 were applied to explore the efficacy of PSCA and MUC1 CAR-T cells against NSCLC. Tumor growth was substantially inhibited in CAR-PSCA T cells. Thereafter, a combination of PSCA and MUC1 CAR-T cells exerted a synergistic effect on tumor survival (104). Therefore, MUC1 and PSCA could be promising CAR-T cell therapy targets for the treatment of NSCLC.

#### 1.3.5 Human Epidermal Growth Factor Receptor 2 (HER2)

HER2 belongs to the HER/ErbB family of receptor tyrosine kinases involved in cell proliferation and angiogenesis (105). The anti-tumor effect of HER2 CAR-T cells against two NSCLC cell lines, A549 and H1650, was observed in a 96-h co-culture assay (106). Moreover, in orthotopic or subcutaneous A549 NSCLC mouse xenograft models, HER2 CAR-T cell therapy decreased tumor growth and could not completely eliminate tumors (106, 107).

#### 1.3.6 Carcinoembryonic Antigen (CEA)

CEA is an oncofetal glycoprotein generally expressed during fetal development; however, its expression declines after birth (108). CEA levels increase rapidly in the tumorigenesis and development of lung cancer (109). Therefore, preclinical studies of CAR-T cell therapy targeting CEA have been conducted. CEA-targeted CAR-T cells have been found to eradicate advanced lung carcinomas (110).

#### 1.3.7 PD-L1

Immunotherapy targeting programmed death-1(PD-1)/PD-L1 signaling has achieved substantial progress in NSCLC treatment. Accumulating evidence shows that PD-L1, both in tumor cells and in the TME, suppresses T cell proliferation and mediates anti-tumor immunity (111). PD-L1-targeted CAR-T cells exhibited robust cytotoxic effects against NSCLC cells *in vitro* and *in vivo* (112, 113). Therefore, PD-L1-targeted CAR-T cells could be a novel curative approach for PD-L1-positive NSCLC patients.

#### 1.3.8 Fibroblast Activation Protein (FAP)

FAP is a marker expressed on cancer- associated fibroblasts (CAFs) in a majority of human malignancies (114). FAP molecule itself and FAP-positive cells in TME could contribute to cancer cell proliferation, invasion, angiogenesis and extracellular matrix (ECM) remodeling (115).

FAP targeted CAR-T cells inhibited the proliferation of TC1 and A549 lung cancer cells by eliminating FAP-positive stromal cells in mice models (114, 116). In contrast, another study claimed that FAP targeted CAR-T cell achieved limited antitumor efficacy and severe side effects for bone marrow stromal cells (BMSCs) were also being killed (117). Therefore, the feasibility of targeting FAP as a specific antigen in CAR-T therapy remains to be verified.

#### 1.3.9 Other Targeted Antigens

**Figure 3:**
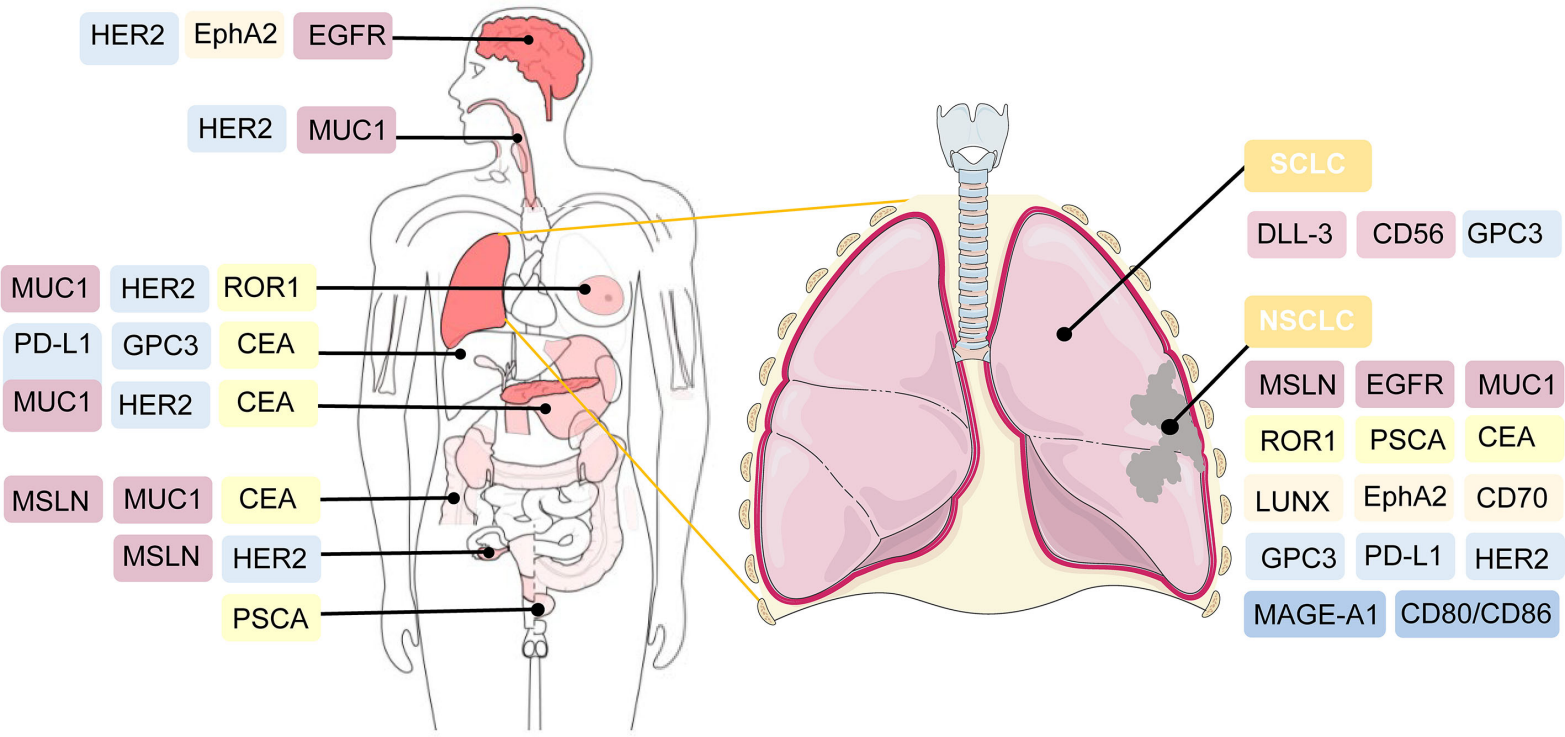
Potential targeted antigens for CAR-T cell therapy in preclinical and clinical trials. In the right, antigen targets are listed against SCLC and NSCLC. As shown in the left of the figure, these antigens are also broadly applied in CAR-T cell therapy against other solid tumors.

Several tumor antigens, such as lung-specific X (LUNX), variant domain 6 of CD44 gene, melanoma-associated antigen-A1 (MAGE-A1), erythropoietin-producing hepatocellular carcinoma A2 (EphA2), and glypican-3 (GPC3), are under active investigation for application as targeted antigens of CAR-T cell therapy against NSCLC (118–122). For SCLC, CD56-and Delta-like ligand 3 (DLL-3)-targeted CAR-T cells are being explored (123, 124). Bivalent tandem CAR-T cells are equipped with two targeted antigens. CD70, B7-H3, MUC1, PSCA, PD-L1, and CD80/CD86, have exhibited enhanced antitumor efficacy in lung cancer (104, 125). B7-H3 is one of inhibitory ligands, which belongs to B7 immunoglobulin family. Although its corresponding immune checkpoint receptors remain undetermined, the inhibitory role of B7-H3 has been confirmed in preclinical studies (126). The expression of B7-H3 is aberrantly augmented in a wide range of solid tumor tissues, compared with normal tissues, which supports the possibility of targeting B7-H3 in CAR-T cell therapy against lung cancer (125, 127). CD80/CD86 are immune checkpoint ligands shared by inhibitory CTLA-4 and costimulatory CD28. CD80/CD86-targeted CAR-T cells have been generated to reverse the inhibitory CTLA4-CD86/CD86 signals and prevent the survival of B cell malignancies and other tumors including NSCLC (128). The efficacy of CAR-T cell therapy, which targets both tumor cells and tumor-associated macrophages in the TME, has also been validated in NSCLC (129) (**Figure 3**).

### 1.4 NSCLC and SCLC−Associated Antigens for CAR−T Cell Therapy in Clinical Trials

CAR-T cell treatment has achieved substantial success against several hematological malignancies. At present, the primary task is to broaden the applications of CAR-T cell therapy from merely hematologic tumors to multiple solid tumors. Thus, its safety and efficacy in solid cancers are under intensive investigation. The feasibility of CAR-T therapy against solid tumors is currently being evaluated in approximately one-third of CAR-T clinical trials (130). Among them, the majority are on CAR-T therapy for the treatment of lung cancer. The extraordinary progress of CAR-T therapy for lung cancer is promising; however, many challenges and hurdles exist. Therefore, the clinical application of CAR-T in NSCLC and SCLC treatment is still under intensive exploration. The optimal target for CAR-T cell therapy is specifically expressed or generally overexpressed in tumor cells, whereas it is expressed at very low or limited levels in normal peripheral cells or tissues (131). Current clinical trials of CAR-T therapy against NSCLC and SCLC primarily focus on MSLN, MUC1, GPC3, PSCA, EGFR, CEA, HER2, PD-L1, ROR1, and other promising targets **(** **Table 2** **)**.

**Table 2:** Underlying targeting antigens of NSCLC and SCLC for CAR-T cell therapy in clinical trials.

Clinical Trial	Cancer type	Targeting antigen	Sponsor	Estimated Enrollment	Phases	Status
NCT03054298	NSCLC	Mesothelin	University of Pennsylvania	18	Phase 1	Recruiting
NCT03330834	NSCLC	PD-L1	Sun Yat-sen University	1	Phase 1	Terminated
NCT04489862	NSCLC	αPD1, MSLN	Wuhan Union Hospital, China	10	Early Phase 1	Recruiting
NCT03392064	SCLC	delta-like protein 3 (DLL3)	Amgen	6	Phase 1	Suspended
NCT03198546	SCLC	GPC3	Second Affiliated Hospital of Guangzhou Medical University	30	Phase 1	Recruiting
NCT04348643	Lung cancer	CEA	Chongqing Precision Biotech Co., Ltd	40	Phase1/2	Recruiting
NCT04864821	Lung cancer	CD276 (B7-H3)	PersonGen BioTherapeutics (Suzhou) Co., Ltd.	24	Early Phase 1	Not yet recruiting
NCT03740256	Advanced HER2 Positive lung cancer	HER2	Baylor College of Medicine	45	Phase 1	Recruiting
NCT02706392	NSCLC	ROR1	Fred Hutchinson Cancer Research Center	60	Phase 1	Recruiting
NCT03525782	NSCLC	MUC1, PD-L1	The First Affiliated Hospital of Guangdong Pharmaceutical University	60	Phase1/2	Recruiting
NCT02587689	NSCLC	MUC1	PersonGen BioTherapeutics (Suzhou) Co., Ltd.	20	Phase1/2	Unknown
NCT04025216	NSCLC	TnMUC1	Tmunity Therapeutics	112	Phase 1	Recruiting
NCT03198052	Lung cancer	HER2, Mesothelin, PSCA, MUC1, Lewis-Y, GPC3, AXL, EGFR, Claudin18.2, or B7-H3	Second Affiliated Hospital of Guangzhou Medical University	30	Phase 1	Recruiting
NCT03060343	NSCLC	PD-L1, CD80/CD86	Yu Fenglei	10	Phase 1	Unknown

## 2 Challenges and Engineering Strategies

Over the past few years, there has been a rapid increase in the use of CAR-T cell therapy to treat hematological malignancies and solid tumors. Many clinical trials have made substantial achievements; however, severe therapeutic responses to CAR-T cell therapy and unsatisfactory treatment efficacy hinder rapid development. In 2010, a patient with multiple metastases of colon cancer died after administering CAR-T cells targeting ERBB2. The patient experienced respiratory distress within 15 min after CAR-T cell transfusion and died five days after the treatment (132). Compared with hematological malignancies, solid tumors face a unique set of challenges, including issues confusing hematological malignancies, more severe and complicated related toxicities, the lack of a strongly expressed tumor-associated antigen target, low infiltration of T cells in tumor tissue, CAR-T cell exhaustion, and a highly immunosuppressive and metabolically challenging TME, which limit the safety and effectiveness of treatment (133–135). Future studies to develop practical engineering strategies to enhance the efficacy of CAR-T cell therapy and minimize adverse reactions should be conducted.

### 2.1 Overcoming Treatment-Related Toxicities

CAR-T cell therapy can result in a range of toxicity events. The major treatment-related toxicities include cytokine release syndrome (CRS) and immune effector cell-associated neurotoxicity (ICANS), which particularly peak in the first or second week of CAR-T cell administration, respectively (133). Patients with CRS mostly have common manifestations such as fever, tachycardia, hypoxia, dyspnea, hypertension, coagulopathy, and elevated serum cytokines, including interleukin-6 (IL-6) (136, 137). ICANS is characterized by tremor, encephalopathy, cerebellar alteration, or seizures (138). Both CRS and ICANS are caused by the activation of CAR-T cells and cytokines secreted by the associated immune cells. CAR-T cells can release pro-inflammatory cytokines, including IL-2, IL-6, and IFN-γ, and then activate more immune cells to secrete IL−1RA, IL−10, IL−6, IL−8, IFNα, and other cytokines, which eventually could lead to massive cytokine release (139). Hemophagocytic lymphohistiocytosis/macrophage activation syndrome has also been reported following CAR-T cell therapy. It is characterized by hyperinflammatory syndrome and multiple organ dysfunction (140). IL−6/IL−6R antagonists and corticosteroid usage can interrupt the inflammatory process and play a substantial role in symptom remission (141). It is critical to detect these treatment-related toxicities early and provide appropriate treatment based on the toxicity grade as soon as possible.

**Figure 4:**
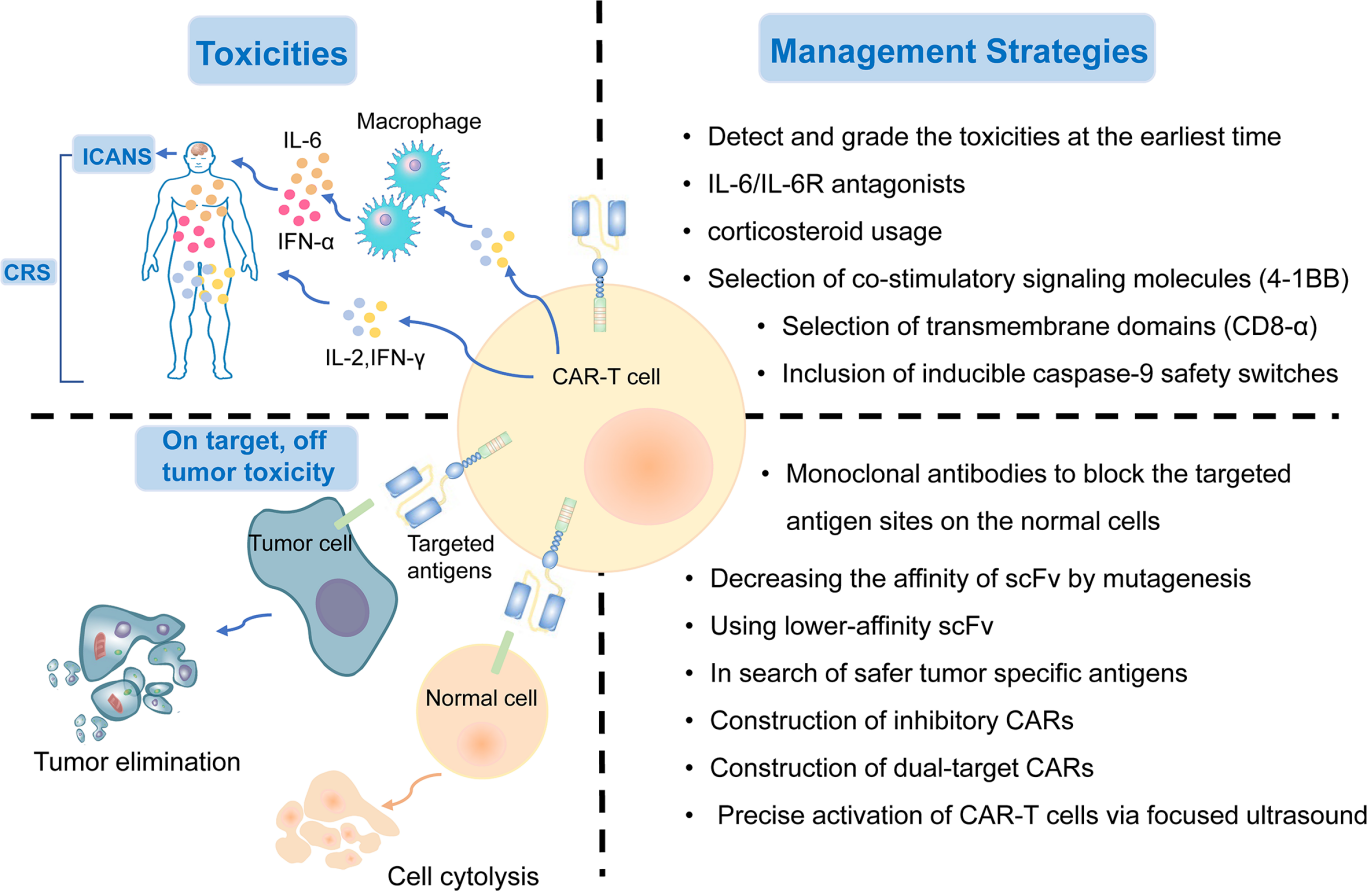
The treatment-related and on-target, off-tumor toxicities and corresponding management strategies of CAR-T cell therapy.

Selecting co-stimulatory signaling molecules and transmembrane domains could have an impact on cytokine production and CAR-T cell function. Compared with CD28/CD3ζ CAR T cells, 4–1BB/CD3ζ CAR T cells amplified more slowly, persisted for a longer time, and secreted less cytokines (142). CAR-T cells with CD8-alpha transmembrane domains have been shown to release less cytokines than those with CD28 domains (64). In addition, the inclusion of inducible caspase-9 safety switches to CARs has been verified to control the expansion of CAR-T cells and the load of cytokines (143). In summary, genetic modification of CAR designs might help reduce the generation of cytokines and the incidence of treatment-related toxicities (**Figure 4**).

### 2.2 On-​Target, Off-​Tumor Toxicity

Although the targeted tumor-associated antigens are carefully screened, many normal cells still suffer from the attack of T cells because they express the same or similar antigens. On-target, off-tumor toxicity, manifesting multiple organ injury and failure, is an issue impeding the development of CAR-T cell treatment. Thus, there is an urgent need to explore safer targeted tumor-associated antigens for lung cancer treatment. To date, MSLN, EGFR, ROR1, MUC1, PSCA, and HER2, as described previously, are the most targeted antigens in CAR-T cell therapy for NSCLC. Several other tumor antigens, including LUNX and B7-H3, also exhibit great potential as targeted antigens in CAR-T cell therapy because they are aberrantly expressed in lung cancer tissues, with a relatively low expression in normal tissues (118, 144).

The on-target toxicity is antigen-oriented, and shielding of a CAR-targeted antigen expressed on normal tissues could minimize toxicity and optimize the efficacy of CAR-T cell therapy. Some renal cell carcinoma patients developed hepatic enzyme disorders that required discontinuation of therapy after receiving anti-carbonic anhydrase IX (CAIX) CAR-T cell therapy. This on-target toxicity can be overcome by pre-administration of parental anti-CAIX monoclonal antibodies to block the CAIX antigen sites in the liver (145). In addition, decreasing the affinity of scFv by mutagenesis or using lower-affinity scFv as a replacement could also substantially reduce on-target, off-tumor reactivity without affecting the antitumor activity (45). Other attempts include the construction of inhibitory CARs, which could protect the normal cells from being attacked by targeted CAR-T cells, and dual-target CAR-T cells, which require two signals to be full activated (146). Recently, an inducible CAR-T cell, was developed to be activated *via* focused ultrasound within specific tumor sites, which could dramatically mitigate the on-target, off-tumor toxicity, in comparison to conventional CAR-T cells (147) (**Figure 4**).

### 2.3 Evasion of Antitumor Immune Responses

A common mechanism for tumor cells to evade immune surveillance in CAR-T cell therapy is the downregulation or even loss of targeted antigens, whose expression level could exert a direct impact on the therapeutic efficacy (148). Targeting CD19/CD20 CAR-T cell therapies have led to promising achievements in treating B-cell malignancies in recent years (149). Tumor-associated antigens in hematologic malignancies are highly expressed and easier to target, whereas antigens in solid tumors have greater heterogeneity and lower expression levels, making it difficult to eliminate solid tumor cells (150). Intratumor heterogeneity might be a key factor contributing to the evasion of antitumor immune responses (151). In lung cancer, common targets such as MSLN, MUC1, PSCA, and epithelial cell adhesion molecule, have intratumoral heterogeneity, leading to an unsatisfactory outcome of CAT-T cell therapy in lung cancer (21). Many clinical studies have shown that when tumors relapse after treatment, tumors are found to undergo antigen loss or become antigen-negative (50, 152). This phenomenon may be mediated by the selective pressure applied by CAR-T cells to tumor cells, leading to the progressive selection of antigen-negative cells (82).

**Figure 5:**
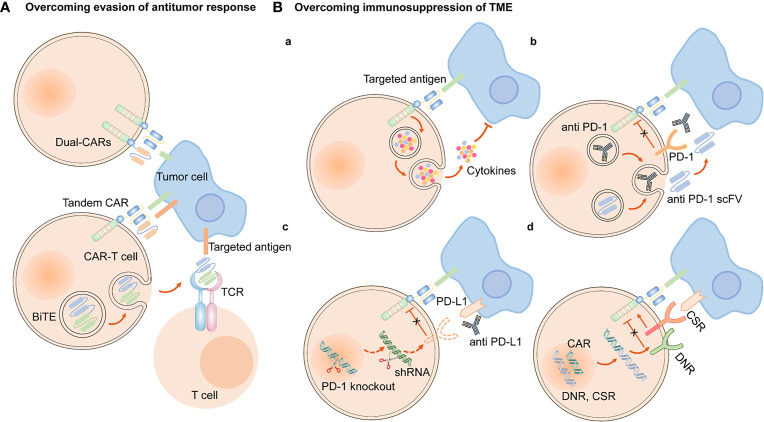
Engineering strategies to overcome evasion of antitumor response and immunosuppression of TME. **(A)** CAR-T cells are engineered to simultaneously target two antigens (dual CAR-T cells), and secret BiTE to redirect both T cells and CAR-T cells against specific tumor cells and circumvent antigen escape. Tandem CAR-T cells have bispecific receptors, which could target two different antigens. **(B) (a)** Armored CAR-T cells expressed immunostimulatory cytokines. Approaches to overcoming the immunosuppression of immune checkpoints in TME are as follows, **(b)** CAR-anti-PD-1/PD-L1 antibodies or scFv, **(c)** PD-1 gene knockout or downregulation of PD-1 expression by shRNA, **(d)** express a PD-1 DNR or a PD-1 CSR.

To overcome the evasion of antitumor immune responses, one approach is to engineer CARs with dual-specificity (i.e., simultaneously targeting two antigens) (153). Bispecific T cell-engagers (BiTEs), consisted of two scFvs, are produced by genetically engineered CAR-T cells to redirect both T cells and CAR-T cells against specific tumor cells (154, 155). EGFRvIII-specific CAR-T cells secreting BiTE have shown to circumvent antigen escape in glioblastoma, and its effect on lung cancers remains to be further investigated (154). Tandem CAR-T cells can mitigate antigen escape and translate into superior antitumor activity (156, 157) (**Figure 5**). Armored CAR-T cells secreting pro-inflammatory cytokines, such as IL-18, have also been shown to elicit an enhanced antitumor immune response in preclinical models (158).

### 2.4 Physical Barriers

Cancer-associated fibroblasts (CAFs) and fibrotic environment contribute to the formation of physical barrier, preventing the CAR-T cells from being trafficked into tumor sites. Less infiltration of CAR-T cells into tumor tissues is another reason why the efficacy of CAR-T cell therapy in NSCLC is not as ideal as that in hematological malignancies.

#### 2.4.1 CAFs

**Figure 6:**
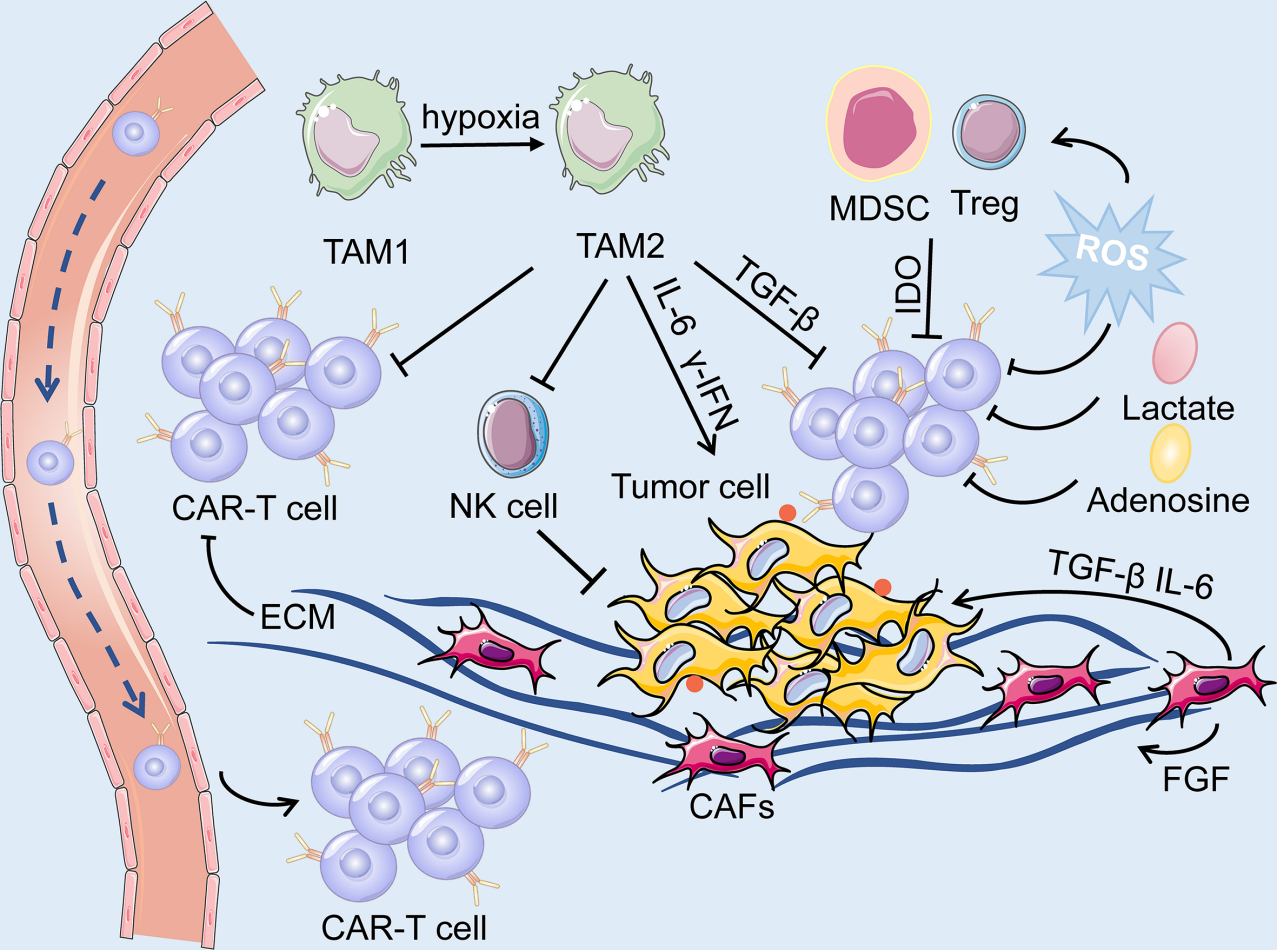
The TME is primarily composed of tumor cells, immune cells, immunosuppressive cells (including TAM, MDSC and Treg) and cytokines, CAFs, ECM and dysregulated tumor vasculatures. On the one hand, ECM produced by CAFs forms a physical barrier, impairing the infiltration of the CAR-T cells. On the other hand, the soluble cytokines secreted by the CAFs mediate immuno-suppressive responses, and consequently, facilitate the survival of tumor cells. The hypoxic and acidic environment directly deteriorate the metabolism of T cells while activating suppressive Tregs, leading to immunosuppression of CAR-T cells.

CAFs are the predominant component of stromal cells in the TME and cannot be cleared by apoptosis (159). Owing to the heterogeneity of CAFs, they could play a dual role in pro-tumorigenicity and anti-tumorigenicity (160). They could regulate the growth, invasion, and angiogenesis of tumor cells by reshaping the ECM and secreting soluble growth factors (160). Moreover, growth factors, cytokines and chemokines, including fibroblast growth factor (FGF), TGF-β, C-X-C motif chemokine ligand 12 (CXCL12), and IL-6, are also secreted by CAFs to mediate immunosuppressive responses (161). Hence, they can be applied as potential targets for anticancer treatment. However, many challenges still prevail in modulating CAFs as an ideal target for CAR-T cell therapy. As previously mentioned, FAP-targeted CAR-T cell therapy induced lethal adverse effects because CAR-T cells attacked FAP-positive BMSCs (117). In addition, CAFs have been shown to contribute to the development of therapeutic resistance because the ECM produced by CAFs could serve as a thick barrier to block the penetration of drugs (162). Accordingly, we hypothesized that the physical barrier formed by CAFs could also hinder the delivery of CAR-T cells into tumor tissues, thus diminishing the effectiveness and efficacy of CAR-T cell therapy (**Figure 6**).

Several studies have been made to deplete or remodel the CAFs in the TME. One potential strategy is to apply FAP-redirected synthetic Notch CAR T cells or heparanase-modified CAR-T cells to deliver CAF remodeling molecules to suppress the expression profile of CAFs (163).

#### 2.4.2 Fibrotic Environment

In contrast to hematological tumors, the infiltrative ability of CAR-T cells in lung cancer tissues is greatly restrained by the presence of a physical barrier. CAF activation, abnormal dense collagen, and ECM deposition contribute to developing a dense and fibrotic environment, altering the localization and migration of effector immune cells in NSCLC, which hinders immune cell infiltration and influences the efficacy of immunotherapy (164, 165). In addition, the extensive fibrotic environment mostly lacks blood vessels, which creates a hypoxic TME and further impairs immune function (166).

The binding of chemokines and their corresponding receptors can mediate the trafficking of CAR-T cells through fibrotic environment. Hence, one approach to enhance the infiltration level of CAR-T cells is to engineer them to express chemokines or transgenic chemokine receptors (167). The CAR-T cells engineered to express IL-7 and CCL19 have been validated to increase the infiltration of peripheral CAR-T cells and dendritic cells and into tumor tissues and enhance the anti-tumor immune responses (168). Another engineering strategy is to construct enzyme-modified CAR-T cells to express heparanase, which accelerates the degradation of ECM and facilitates CAR-T cell trafficking to tumor sites (169). In addition, local injection of CAR-T cells is under investigation.

### 2.5 Immune Suppression in the TME

The TME of lung cancer has an immunosuppressive effect, as T cell activity is suppressed due to anti-inflammatory cytokines and upregulated immune checkpoint ligands. Additionally, the immunosuppressive cells, such as myeloid-derived suppressor cells (MDSCs), regulatory T cells (Tregs), tumor associated macrophages, and tumor associated neutrophils are broadly present in the TME (**Figure 6**). CAR-T cell therapy against lung cancer is less efficient because of immune suppression of the TME and loss of CAR-T cell function.

One engineering approach to overcome the immunosuppressive role of TME is to establish armored CAR-T cells that secrete pro-inflammatory cytokines or chemokines, such as IL-12, IL-15, and IL-18 (20). These cells can recruit and activate innate immune cells such as natural killer (NK) cells and macrophages, and reprogram the immunosuppressive TME, which subsequently supports the proliferative and antitumor activity of CAR-T cells (170). In addition, based on blocking immune checkpoints, genetic knockdown of immune checkpoint receptors in CAR-T cells, such as PD-1, was demonstrated to enhance the anti-tumor effect. The clinical outcomes are being actively assessed in clinical trials on lung cancer (171). Other strategies include engineering CAR-T cells to secrete immune-checkpoint inhibitors, including anti–PD-1 scFv and anti–PD-L1 antibodies, to express PD-1 dominant-negative receptors (DNR) or PD-1 chimeric switch receptors (CSR) (113, 172, 173) (**Figure 5**).

### 2.6 Metabolic Profile of the TME

Cumulating evidence supports that metabolism plays an essential role in the immune response because it could regulate the function and activity of T cells. The inhibition of T cell metabolism may directly deteriorate the activity of T cells while activating suppressive Tregs, resulting in immuno-suppression (174). The proliferation of CAR-T cells, secretion of cytokines, and elimination of tumor cells are all energy-demanding processes. However, tumor cells mostly consume a large proportion of energy and nutrients, while generate a mass of immunosuppressive metabolites, such as adenosine, lactate, and kynurenine (135, 174). Moreover, indolamine-2,3-dioxygenase (IDO) secreted by tumor cells and MDSC could catalyze tryptophan into kynurenine, leading to the inactivation of CAR-T cells and the proliferation of Tregs (175) (**Figure 6**). On the other hand, the dysregulated vasculatures also result in an extremely hypoxic and acidic TME. All of the above elements contribute to the formation of the metabolically hostile TME, which further impairs the function of CAR-T cells.

Reprogramming the CAR-T cells to adjust their metabolic properties through genetic or pharmacological inhibition of adenosine receptors A_1_ and A_2A_R substantially elevated CAR T cell efficacy in breast cancer, which appears to be a promising method to enhance CAR-T cell function in the TME (176). Additionally, ROS generated by MDSC exerts a negative impact on CAR-T cells, and therefore, the reduction of ROS might be a potential strategy to overcome the metabolic profile of TME. Furthermore, CD28 and 4-1BB, the co-stimulatory domains of CAR-T cells, respectively, improved the metabolic fitness of CAR-T cells in melanoma by upregulating the intake of glucose and the expression of glycolytic enzymes, and enhancing mitochondrial biogenesis and oxidative metabolism (177, 178). However, limited data are available on the metabolic reprogramming of CAR-T cells in lung cancer.

### 2.7 CAR-T Cell Exhaustion

The existence of inhibitory ligands in the TME and endogenous TCRs leads to the gradual exhaustion of CAR-T cells (134). Clinical evidence has confirmed that CAR-T cell exhaustion markedly limits the efficacy of CAR-T cell therapy; therefore, it is imperative to prevent or reduce CAR-T cell exhaustion. However, it is difficult to reverse the cell exhaustion process directly by dedifferentiating T cells for exhaustion, which is a transcriptional and epigenetic forced differentiation state (179). Therefore, less differentiated T cell populations, such as naive T cells, whose proliferative activity is more robust, are selected for CAR-T cell manufacture (180). The negative regulators inducing T-cell exhaustion include PD-1, CTLA4, T-cell immunoglobulin and mucin domain 3, and lymphocyte-activation gene 3, which could restrain the activity of T cells while promoting the suppressive function of Tregs (181–183).

The above research advancements may shed light on new strategies to increase CAR-T cell persistence. Engineering strategies to inhibit these negative regulators primarily involve: (1) immune checkpoint blockades, (2) genetic knockdown of negative regulators in CAR-T cells, (3) PD-1 DNR, and (4) autocrine secretion of anti–PD-1 scFv and anti–PD-L1 antibodies from CAR T cells (20, 73, 182, 184). At present, combination therapy of CAR-T cells and immune checkpoint blockades has been utilized to overcome CAR-T cell exhaustion in clinical trials of NSCLC (185). CRISPR/Cas9-mediated knockdown of negative regulators in CAR-T cells may become a novel therapeutic approach to increase the persistence of CAR-T cells (182). CAR-T cells targeting PD-L1z, equipped with CAR-T cells with intrinsic blockade properties of PD-1, demonstrated efficacious antitumor activity in NSCLC models (113). CAR-T cells secreting anti–PD-L1 antibodies have been demonstrated to combat T cell exhaustion in a renal cell carcinoma mouse model (172) (**Figure 5**). In addition, transient cessation of CAR signaling, 4-1BB and CD28 costimulatory signaling, c-Jun, and transcription factors, such as nuclear receptor subfamily 4 group A, NFAT, and thymocyte selection-associated high mobility group box protein have also been shown to regulate T cell exhaustion (179, 184, 186). Further studies are required to apply these findings to enhance CAR-T cell resistance to exhaustion.

## 3 Future Outlook

**Figure 7:**
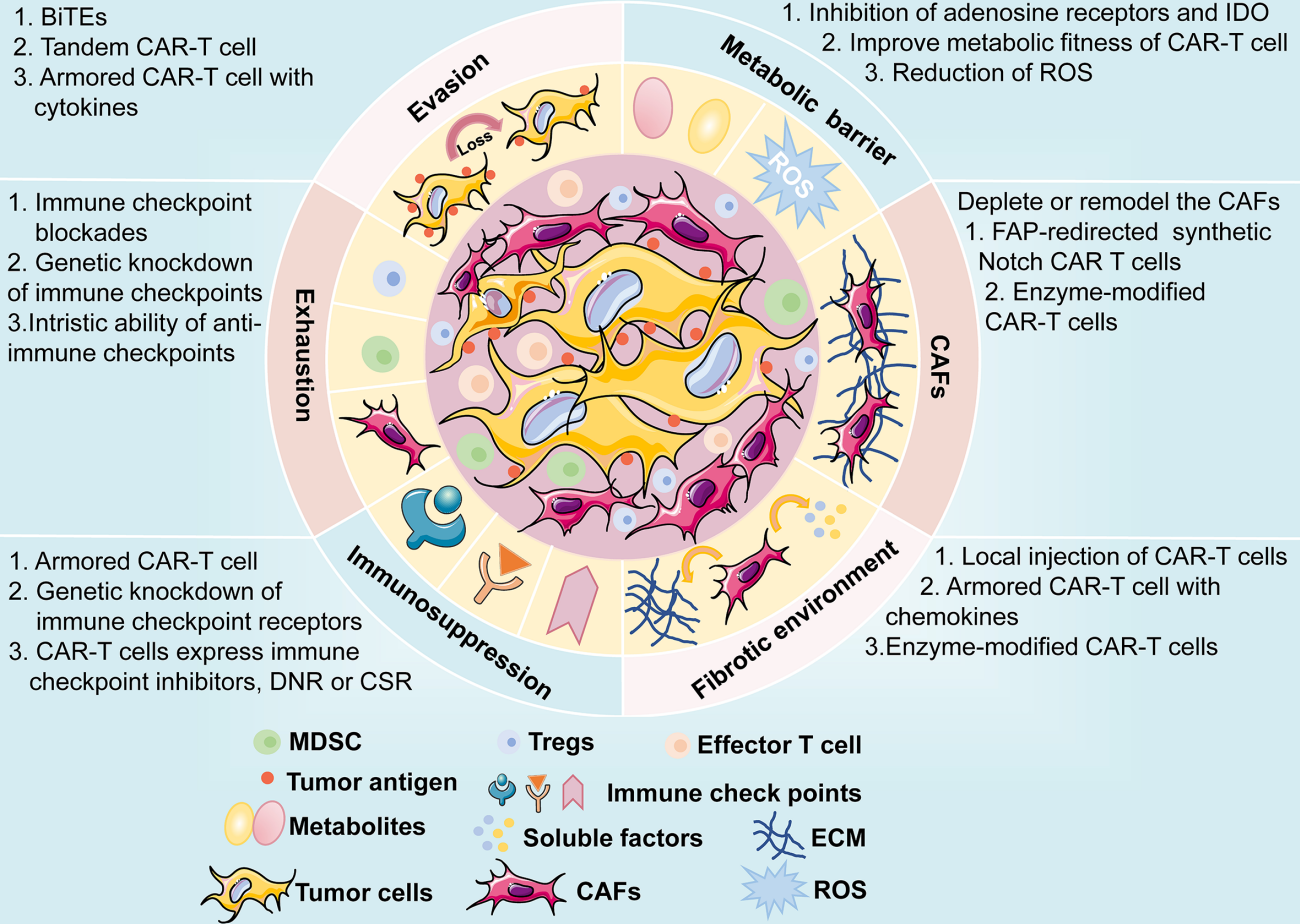
A brief overview of potential challenges faced by CAR-T cell therapy, including antigen evasion, metabolic barrier, CAFs, fibrotic environment, immunosuppression of TME, and exhaustion of CAR-T cells. The possible mechanisms and engineering strategies are also presented.

CAR-T cell therapy has emerged as a novel and effective immunotherapy against multiple cancers, especially hematological malignancies. The same issues, such as CAR-T therapy-related toxicities, on-target, off-tumor toxicity, and evasion of antitumor responses, have plagued the treatment of hematologic malignancies; the treatment of solid tumors encounters even greater challenges. Moreover, the physical barrier impedes the infiltration of CAR-T cells to tumor sites, and the TME is immunosuppressive. In recent years, the successful improvements in the safety and efficacy of the therapy have facilitated the application of CAR-T therapy in solid tumors, including lung cancer. CAR structures persistently undergo evolution to enhance efficacy and reduce the cytotoxic effects of CAR-T cell therapy. In addition, the engineering solutions mentioned above are in their early stages and are being progressively developed towards the clinical application phase, and further investigations are expected **(** **Figure 7** **)**. Among these engineering strategies, gene editing technology is one of powerful tools to improve the efficacy and safety of CAR-T cell therapy and is driving the application of this novel cancer therapy. The manufacture of “off the shelf” CAR-T cell products by disrupting the TCR alpha/beta chains through TALENs or CRISPR/Cas9 platform, is currently undergoing the evaluation of clinical trials (187). The inclusion of inducible caspase-9 safety switches to CARs could regulate the production of cytokines to prevent CRS (143). CRISPR/Cas9-mediated knockdown of negative immune checkpoints enables the CAR-T cells to resist the immunosuppressive TME. It is too early to appreciate the promising prospects of this novel immunotherapy approach in lung cancer treatment until more clinical trials to investigate these engineering strategies are conducted and evaluated.

## Author Contributions

B-TY and NW contributed significantly to fund support and the conception of the review. B-FX, J-TZ and Y-GZ contributed to wrote the manuscript. X-RC contributed to make preparations and revise the manuscript. Z-ML helped proposed some constructive suggestions. All authors contributed to the article and approved the submitted version.

## Funding

This work was supported by the National Key Research and Development Program of China (No. 2018YFC0910700), Beijing Human Resources and Social Security Bureau (Beijing Millions of Talents Project, 2018A05), Beijing Municipal Administration of Hospitals’ Youth Programme (QMS20191107), National Natural Science Foundation of China (No. 81972842), Beijing Natural Science Foundation (No. 7192036), Natural Science Foundation of Jiangxi Province (20202BABL206088).

## Conflict of Interest

The authors declare that the research was conducted in the absence of any commercial or financial relationships that could be construed as a potential conflict of interest.

## Publisher’s Note

All claims expressed in this article are solely those of the authors and do not necessarily represent those of their affiliated organizations, or those of the publisher, the editors and the reviewers. Any product that may be evaluated in this article, or claim that may be made by its manufacturer, is not guaranteed or endorsed by the publisher.
